# Blockchain security enhancement: an approach towards hybrid consensus algorithms and machine learning techniques

**DOI:** 10.1038/s41598-024-51578-7

**Published:** 2024-01-11

**Authors:** K. Venkatesan, Syarifah Bahiyah Rahayu

**Affiliations:** 1 Cyber Security & Digital Industrial Revolution Centre, National Defence University Malaysia (UPNM), Kuala Lumpur, Malaysia; 2 Present Address: Department of Science Defense, Faculty of Defense Science and Technology, National Defence University Malaysia (UPNM), Kuala Lumpur, Malaysia

**Keywords:** Engineering, Mathematics and computing

## Abstract

In this paper, we propose hybrid consensus algorithms that combine machine learning (ML) techniques to address the challenges and vulnerabilities in blockchain networks. Consensus Protocols make ensuring agreement among the applicants in the distributed systems difficult. However, existing mechanisms are more vulnerable to cyber-attacks. Previous studies extensively explore the influence of cyber attacks and highlight the necessity for effective preventive measures. This research presents the integration of ML techniques with the proposed hybrid consensus algorithms and advantages over predicting cyber-attacks, anomaly detection, and feature extraction. Our hybrid approaches leverage and optimize the proposed consensus protocols' security, trust, and robustness. However, this research also explores the various ML techniques with hybrid consensus algorithms, such as Delegated Proof of Stake Work (DPoSW), Proof of Stake and Work (PoSW), Proof of CASBFT (PoCASBFT), Delegated Byzantine Proof of Stake (DBPoS) for security enhancement and intelligent decision making in consensus protocols. Here, we also demonstrate the effectiveness of the proposed methodology within the decentralized networks using the ProximaX blockchain platform. This study shows that the proposed research framework is an energy-efficient mechanism that maintains security and adapts to dynamic conditions. It also integrates privacy-enhancing features, robust consensus mechanisms, and ML approaches to detect and prevent security threats. Furthermore, the practical implementation of these ML-based hybrid consensus models faces significant challenges, such as scalability, latency, throughput, resource requirements, and potential adversarial attacks. These challenges must be addressed to ensure the successful implementation of the blockchain network for real-world scenarios.

## Introduction

The Consensus protocols employed in the blockchain network provide high security and more efficient operations. Hybrid consensus algorithms are developed by combining the key elements of various consensus algorithms. This might be useful to prevent double-spending and 51% of attacks. Combining Proof of Work (PoW) and Delegated Proof of Stake (DPoS) improves computation performance and enhances high security^1^. DPoS is used for block validation, and PoW is used for block creation, making it more difficult for an attacker to control the network. The combination of Proof of Stake (PoS) and Proof of Work (PoW) results in better security performance along with the network's decentralization^2^. PoS is used for block validation, and PoW is used for block creation, increasing the network's security and decentralization^3^. Integrating the DPoS and Practical Byzantine Fault Tolerance (PBFT) provides higher security, scalability, and efficiency. Here, DPoS is used for block creation, and PBFT is used for block validation, providing better security and scalability^4^.

**Figure 1:**
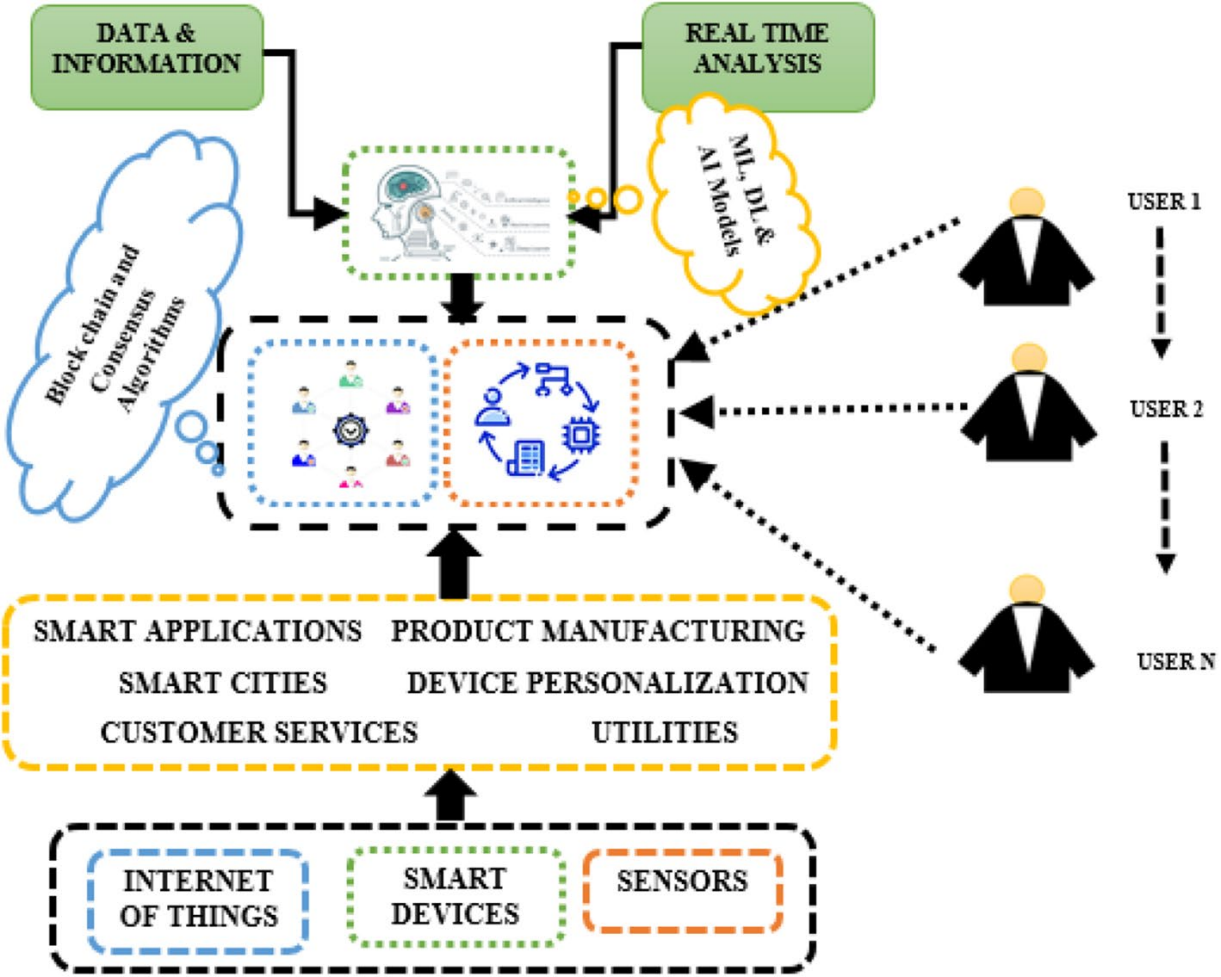
Basic block diagram using blockchain and ML techniques.

Hybridization of the Casper and PBFT consensus algorithms can provide a higher level of security against 51% of attacks in blockchain technology. Casper Algorithm adopts PoS for block validation^5^. Here, validators are selected based on their network stake size. PBFT works differently compared to other algorithms. It confides a group of validators to grasp the following block consensus. Hybridization of PoS and PBFT leverages high-level security and rapid consensus time. This makes it very difficult for attackers to access the network and perform cyber-attacks. In addition, consensus hybridization balances scalability and decentralization, which is convenient for microgrid networks^6^. For any algorithm, achieving a complete security audit and system update, which confirms the efficacy, is essential. Compared to other individual consensus algorithms, these hybridization algorithms enhance security by preventing 51% of attacks^7^. However, analyzing the trade-offs before implementing them in blockchain networks is mandatory. Figure 1 shows the basic block diagram using the blockchain network and ML techniques.

Many researchers propose a new consensus protocol that performs better security and scalability and reduces the probability of cyber-attacks on the blockchain network. In^8^, the author proposed Algorand, which achieves transaction finality and high scalability^9^ introduces consensus algorithms, which enhance throughput, scalability, and security. The authors Zhu^10^ introduced the GHOST protocol, the modified PoW consensus protocol, which achieves the best security and high throughput transaction. Meanwhile^11^, A. Kiayias et al. propose ouroboros, the modified PoS protocol that enhances scalability and security. Lashkari et al.^12^ eliminate 51% of attacks using Bitcoin-NG, improving throughput and scalability. These works accentuate the necessity of creating new consensus protocols that perform highly scalable security and have the potential to handle vast volumes of transactions.

The Hybrid consensus algorithms with Machine Learning (ML) techniques gained significant approaches, which led to high performance, improved scalability, and enhanced security in blockchain networks. Several researchers, such as Zhang^13^, Andoni^14^, Wang^15^, and Yang^16^, conduct various simulations and experiments and discusses hybrid protocols, which evaluate their efficacy and perform better results and security than the existing consensus mechanisms. Additionally, Mahmood et al.^17^ propose a comprehensive review of consensus protocols that enlighten the potential of ML approaches for enhancing performance and security. The author highlights the hybrid consensus mechanism and ML integration to achieve optimal block validation and improve network performance.

Over the years, the performance of the hybrid consensus protocol combined with Particle Swarm Optimization (PSO) has been studied. In^18^, Zhu et al. propose a PoS-based consensus model with PSO that achieves high scalability, better performance, and enhanced security. Razali et al.^19^ Introduce a new consensus model, which uses PSO for block validation optimization. Ali et al.^20^ Work on hybrid consensus protocol with PSO approaches, which results in high security and scalability and maintains high performance. Ullah et al.^21^ propose ML-based PSO for faster block validation and better throughput. Kumar et al.^22^ Provide a comparative study of existing consensus protocol and highlight the potential of the hybrid consensus protocol with PSO optimization techniques. It also demonstrates the strength of hybridization and achieves better performance and scalability, enlightening future research requirements in blockchain networks.

Unsupervised, supervised, and rule-based ML approaches^23^ are vital in responding to and detecting microgrid attacks in blockchain networks. ML techniques may also be used to solve communication and behavior-based attacks. ML-based hybrid consensus algorithms improve security and other performance factors and provide a scope for active research^24^. Specifically, new consensus protocols with ML approaches detect and prevent the significant threats of attacks in blockchain networks. These papers propose simulation models and demonstrate experiments that can perform better security than any other consensus mechanisms^25^. Additionally, these papers provide a comprehensive overview, realize the potential of hybridization, and highlight the importance of further research to develop more effective and efficient methods to secure blockchain mechanisms^26^.

However, the researchers propose a diverse solution, develop a creative approach, and offer a state-of-the-art framework to overcome cybersecurity issues. In^27^, the author proposes Fed-Inforce-Fusion, which is a reinforcement learning-based fusion model for the Internet of Medical Things (IoMT) networks. This method incorporates federated and reinforcement learning to improve accuracy and detection. Meanwhile, the "PC-IDS" framework uses hybrid machine learning approaches to identify harmful behaviors and secure privacy in cyber-physical power networks^28^. However, deep autoencoder IDS performs well and accurately in real-time intrusion detection in IIoT networks^29^.

Additionally, the multi-stage AV framework combines the state-of-the-art framework and deep learning techniques, outperforming existing systems in understanding and identifying cyber risks within autonomous vehicles^30^. Therefore, the relevance of these studies lies in their exploration of hybrid consensus algorithms and integration of machine learning approaches, offering valuable insights into blockchain and cyber security attacks. Despite their contributions, several limitations were prevalent in the studies reviewed. These limitations underscore the need for further research to address these gaps and refine our understanding of integrating machine-learning approaches in hybridizing consensus algorithms.

### Open challenges and motivation

Consensus algorithms face several open challenges that need to be addressed for the widespread adoption of blockchain technology in real-world applications. One of the primary challenges is **scalability**, as consensus mechanisms must efficiently handle many transactions per second without compromising security and decentralization. Another critical challenge is **energy efficiency**, particularly in Proof of Work (PoW), where the high energy consumption is unsustainable and costly in the long run. Developing energy-efficient consensus algorithms or improving existing ones is vital for practical applications. Ensuring fast transaction confirmation times is another challenge to meeting the demands of real-time applications. Long confirmation times can hinder the usability of blockchain technology, making it necessary to optimize latency and transaction confirmation. **Security** is an ongoing concern, and consensus mechanisms must resist attacks such as double-spending, Sybil, and 51% attacks. Enhancing security measures is crucial for building trust and widespread adoption.

**Governance and compliance** are significant challenges, as consensus algorithms must align with legal and regulatory requirements while maintaining decentralization. Finding the right balance between compliance and decentralization is crucial for the finance, healthcare, and supply chain management industries. Interoperability between blockchain networks and consensus algorithms is essential for collaboration and communication across systems. Developing standards and protocols for seamless integration and data exchange is challenging. **Privacy and confidentiality** are paramount, and consensus algorithms must incorporate robust techniques to protect sensitive data while maintaining transparency and audibility. Consensus mechanisms should **adapt to dynamic networks**, where nodes can join or leave anytime and handle challenges such as node churn, network partitions, and malicious nodes. Improving the user experience is vital for widespread adoption, requiring consensus algorithms to minimize transaction fees, reduce latency, and provide a seamless and intuitive interface.

Addressing the environmental impact of consensus algorithms, particularly energy-intensive ones like PoW, is a pressing challenge. Developing sustainable and eco-friendly consensus algorithms is essential to align blockchain technology with global sustainability goals. Overcoming these challenges will require continuous research, innovation, and collaboration between industry, academia, and regulatory bodies. By addressing these open challenges, consensus algorithms can pave the way for the widespread adoption of blockchain technology in various real-world applications.

### Research gap identification

The analysis of existing literature reveals a distinct gap in integrating hybrid consensus algorithms with machine learning approaches. While previous studies have laid essential groundwork, there remains unexplored territory in understanding the concepts of hybridization and difficulties in integrating machine learning approaches to enhance the security of the blockchain network. This research strives to fill this gap by defining the research objectives, thereby advancing our understanding of improving security in blockchain networks and protecting the network from malicious attacks.

### Research objectives

The main goal of this research paper is to implement a hybrid consensus mechanism with ML techniques, which enhances the security of the consensus mechanisms and avoids cyber attacks. The contribution of this research is listed below.

- To identify and understand the vulnerabilities in existing mechanisms. Here, analyzing and evaluating the security shortcomings enables a comprehensive understanding of the vectors and potential threats.
- To reframe the hybrid consensus Algorithms. A comprehensive analysis of consensus algorithm hybridization and its necessity in cyber security is discussed here.
- To develop the ML framework for extraction of the features and anomaly detection. The critical aspects of the ML framework that effectively performs feature extraction, anomalies, and malicious activity detection within the blockchain network must be performed. This framework will leverage advanced ML algorithms to analyze network behavior, identify suspicious patterns, and distinguish normal activities from potential attacks.
- To integrate the ML framework with consensus mechanisms. The developed ML framework will be integrated into the consensus algorithms discussed in this work to enhance the security of consensus mechanisms. This integration will enable real-time monitoring and proactive defense mechanisms against attacks, thereby ensuring the integrity and stability of blockchain networks.
- To evaluate the effectiveness of the hybrid approach. The proposed hybrid ML approach can be thoroughly evaluated. The evaluation would be focused on security enhancements achieved through the proposed solution, which leads the system to an adoptive selection of consensus algorithms and intelligent decision-making.

By addressing these challenges, this research paper aims to contribute to blockchain security by proposing novel consensus algorithms and hybrid ML approaches that enhance the overall security of blockchain networks and mitigate the risk of cyber-attacks.

The contribution of the proposed hybrid consensus algorithms is listed below.

- This proposed hybridization combines the strength of different consensus mechanisms, mitigates the vulnerabilities, and enhances scalability.
- The hybrid consensus algorithm uses energy-efficient mechanisms to reduce environmental impact without compromising security.
- This model can adapt to dynamic conditions and confirms robustness.
- This proposed method can integrate privacy-enhancing features and protect sensitive information.
- Additionally, hybrid models can improve trust by providing robust and resilient consensus mechanisms.

The contributions of the ML techniques are listed below:

- ML can greatly enhance threat detection capabilities and improve the trustworthiness of the network by providing a multilayer defense.
- It improves the adaptability to emerging threats and acts as an intelligent layer to optimize consensus mechanisms based on network conditions and improve scalability challenges.
- ML techniques can adjust the consensus mechanism based on energy availability and consumption patterns.
- It also learns continuously from the network behavior and adjusts security measures to meet network dynamics.
- Cryptographic techniques and advanced privacy-preserving algorithms can further enhance the confidentiality of transactions and user data.
- Decentralized systems can detect and respond to security threats using machine learning to identify patterns and anomalies, reducing the risk of successful attacks.
- Resource allocation can be optimized by dynamically assessing threat levels and adjusting security measures accordingly. This ensures efficient allocation of resources to areas with the highest risk.

The rest of the paper is organized as follows: Section "Background study" establishes the preliminaries for the challenges and vulnerabilities in existing consensus mechanisms. A detailed discussion of the previous research on 51% of attacks and their impact is also presented in this section. ML Techniques for Security Enhancement are discussed in Section "Machine learning techniques for security enhancement". Section "Research methodology and materials" presents the research methodology and materials and its advantages and optimizations achieved through the proposed approach. The experimental implementations and results are discussed in Section "Security enhancements achieved through the proposed solution". Section "Open issues and challenges of the hybrid consensus approach" presents the proposed hybrid consensus approaches' open challenges and future scope. The conclusions and future work of the paper are discussed in Section "Conclusion".

## Background study

### Challenges and vulnerabilities in existing consensus mechanisms

The progress in blockchain technology has played a crucial role in addressing challenges related to decentralization, security, and consensus formation using various consensus mechanisms. However, these mechanisms have their own set of challenges and vulnerabilities. This section will delve into the concerns and susceptibilities of existing consensus mechanisms.

#### Proof of work (PoW)

PoW is a consensus algorithm that exhibits several challenges and vulnerabilities. Firstly, PoW requires substantial computational power and energy consumption to solve complex cryptographic problems, resulting in high energy consumption that is environmentally unsustainable^31^. This energy-intensive nature raises concerns about the ecological impact of blockchain networks utilizing PoW. Secondly, scalability becomes a concern as the network grows, as the PoW algorithm needs help to maintain efficient consensus and transaction validation in the face of increasing participant numbers^32^. The computational requirements become increasingly demanding, potentially limiting the scalability potential of PoW-based blockchains.

Additionally, the concentration of mining power in a few large mining pools introduces concerns regarding mining centralization and the potential for collusion^33^. This centralization raises questions about the democratic nature of the blockchain network and the potential for malicious activities by a concentrated mining power^34^*.* The priority toward long-term sustainability and PoW-based blockchain adoptions are required to address the challenges.

#### Proof of stake (PoS)

The Proof-of-Stake (PoS) consensus protocols face vulnerabilities and challenges that include the possibility of wealth concentration and must be addressed within the network^35^. In PoS, validators are chosen based on the number of coins they have staked, meaning that those with more enormous stakes are more likely to be selected as validators. This wealth concentration can undermine the network's decentralization and compromise its security^6^. Another issue is the nothing at stake problem, where validators can vote on multiple forks simultaneously without consequences. Validators can create forks and change transaction history, which may lead to double-spending attacks^36^. To mitigate these challenges, solutions such as implementing penalties for malicious behavior, implementing robust governance mechanisms, and ensuring widespread participation can help maintain the security and integrity of the PoS network. Additionally, ongoing research and development efforts are needed to address these vulnerabilities and improve the overall robustness of the PoS consensus mechanism^37^. PoS has energy efficiency advantages, but its vulnerabilities must be acknowledged to ensure blockchain network security.

#### Delegated proof of stake (DPoS)

The DPoS algorithm is widely adopted for its effectiveness and scalability; however, it poses significant challenges and vulnerabilities. A primary concern is the potential for centralization, as DPoS relies on a small group of trusted delegates responsible for block production and validation^38^. This concentration of power introduces the risk of influential entities engaging in vote buying or collusion, compromising decentralization and fairness. Another area for improvement is low voter participation, leading to a lack of representation and potential governance problems^39^. In order to overcome these challenges, measures should be implemented to promote decentralization and encourage greater voter engagement. This includes preventing manipulation through mechanisms that safeguard the integrity of the election process^40^. Educating token holders about the importance of voting and its impact on network governance can increase participation. Introducing mechanisms for delegate rotation or limiting their terms can prevent long-term centralization^41^. Through continuous research and protocol improvements, DPoS can balance efficiency and decentralization, offering a more inclusive consensus mechanism for blockchain networks by enhancing transparency and promoting active participation^42^.

#### Practical byzantine fault tolerance (PBFT)

PBFT offers the advantage of withstanding Byzantine faults but also presents challenges and assumptions that must be addressed. Scalability is a limitation of PBFT, as the latency increases with more nodes due to the communication required for consensus^43^. This makes PBFT more suitable for smaller networks or consortium blockchains. The assumption of PBFT regarding the number of faulty nodes is critical, as it assumes that at most one-third of nodes are faulty. When a higher proportion of nodes become malicious or inaccurate, PBFT's ability to maintain consensus can be compromised^44^. However, researchers have dedicated their efforts to strengthening the scalability and resilience of PBFT in order to overcome these challenges^45^. Optimizations like parallelization and batching have been proposed to reduce communication overhead and latency in more extensive networks. Fault-tolerant algorithms and Byzantine fault detection techniques aim to handle situations where the assumed threshold of faulty nodes is exceeded^46^. Hybrid consensus models combining PBFT with Proof of Stake or Proof of Work have also been explored to balance scalability and fault tolerance. Researchers have proposed various methods to overcome these challenges and improve the scalability, durability, and usability of PBFT in various blockchain networks^47^.

#### Proof of authority (PoA)

PoA relies on a predetermined set of approved validators responsible for validating and adding new blocks to the blockchain. While PoA offers certain benefits, it also poses notable limitations and vulnerabilities. A key concern is the potential for centralization^48^. However, carefully selecting validators and proactive measures to prevent infiltration can ensure continued decentralization and strengthen network security, leading to a robust and resilient system. The concentration of authority in the hands of a small group of validators contradicts the decentralization goal that blockchain technology aims to achieve^49^. Another challenge in PoA is the need for incentives for validators. Unlike Proof of Work and Proof of Stake, PoA does not provide incentives or rewards to validators, as block creation authority is based on reputation or identity rather than a commitment of resources. The absence of incentives will lead to lower participation and highly compromise the network's security^50^. Validators may become less vigilant or refrain from active participation in block validation, leading to a less secure and reliable network. Certain modifications have been proposed for PoA to address these challenges. For instance, implementing a reputation-based system or penalties for misbehavior can mitigate the risk of collusion among validators^51^.

Additionally, offering incentives in the form of transaction fees or token awards can promote active participation and ensure the stability and security of the network. The PoA system offers faster transactions and uses less energy. However, there are concerns about security and trust because it relies on a set of predetermined validators. Additionally, when implementing PoA, it is necessary to consider the balance between centralization and decentralization^50^.

#### Casper

Casper presents a more secure and reliable consensus mechanism, addressing challenges traditional proof-of-stake (PoS) algorithms face. However, a significant challenge in Casper lies in parameter selection. Careful consideration is required to balance security, liveness, and fault tolerance^52^. Improper parameter values can lead to vulnerabilities and compromise the protocol's effectiveness. Conservative parameters may hinder efficient block finalization, while permissive parameters can increase the risk of malicious behavior^53^. Thorough analysis and understanding of network dynamics and trade-offs are necessary to determine appropriate parameter values for Casper. Another critical aspect of Casper is using slashing conditions to penalize validators for malicious behavior^54^. However, defining and enforcing slashing conditions without introducing false positives or negatives is a complex challenge. False positives penalize honest validators wrongly, while false negatives allow malicious validators to escape penalties. Striking the right balance is crucial to prevent unfair penalization and ensure appropriate punishment^55^. Designing robust and accurate slashing conditions requires careful consideration and analysis to minimize false positives and negatives.

Researchers are enhancing Casper to address system challenges through better parameter selection and slashing conditions. This method involves rigorous empirical analysis, simulation studies, and formal verification techniques^56^. It also enables us to confidently determine the most influential parameter choices and optimize the settings for feasible results. Extensive testing and experimentation assess the impact and behavior of slashing conditions in real-world scenarios^57^. Future advancements may involve automated or adaptive parameter selection mechanisms that dynamically adjust based on network characteristics. Similarly, improvements in slashing conditions can be achieved through ML or incorporating external reputation systems. Continued research and development efforts will lead to more robust and practical implementations of Casper.

### Previous research on cyber 51% of attacks and their impact

Previous research on 51% of attacks has explored the potential risks and consequences of these attacks in blockchain networks. By gaining control over 50% of the network's mining power, an attacker can manipulate the blockchain's transactions, potentially leading to double spending or other fraudulent activities*.* It is necessary to assimilate the importance of 51% of attacks for establishing adequate security and authenticity maintenance of the blockchain network.

#### Attack vector identification

This is a challenging research problem related to 51% of attacks. Several studies and experiments have been conducted to understand the various attack vectors. By analyzing these vectors, researchers can enhance security and improve countermeasures. Mining centralization is one of the prominent attacks. Studies are performed to examine the mining power concentration^58^. This study includes investigating the distribution of mining resources, examining the incentives for mining centralization, and identifying the potential risks associated with such centralization. Network partitioning usually occurs when we split the blockchain network into multiple subnets. These results from various technical issues, intentional attacks, and other network disruptions, which blockchain networks need to address^59^. However, researchers have analyzed the influence of network partitioning on consensus mechanisms and their vulnerabilities. After examining these problems, researchers aim to mitigate the issues connected with network partitioning and ensure blockchain integrity. Rent attacks acquire more computational power and control the hash rate's prominence, enabling attackers to manipulate the transaction. Researchers can examine the influence of this attack on other consensus mechanisms, which emphasize the importance of prevention protocols to avoid the presence^20^. However, researchers can also investigate the chance of group mining to launch the attack. When mining pools or entities collude with each other, they combine the computational power and control the hash rate, as discussed in the rental attack, and manipulate the transaction in the blockchain network^60^. This results in the importance of protection mechanisms to avoid and reduce collusion between the mining entities. Furthermore, researchers also analyzed these attacks to detect and mitigate the related vulnerabilities.

#### Double spending and transaction reversal

These attacks aim to achieve double-spending and transaction reversal, exploiting vulnerabilities in the transaction verification process. By controlling most of the network's mining power through a 51% attack, an attacker can manipulate the blockchain's transaction history, allowing them to spend the same coins multiple times^61^. Previous research has extensively explored the economic incentives and feasibility of double-spending attacks, considering factors such as attack cost, potential gains, and impact on the network's reputation^62^. Evaluating the economic viability of these attacks helps understand their motivations and enables countermeasures to be developed. Proposed countermeasures include increasing the number of confirmations required for transaction finality, implementing mechanisms for detecting suspicious transactions, and enhancing consensus algorithm security^63^. Advancements in blockchain technology have introduced additional measures to mitigate double-spending risks, such as faster block confirmation times and additional security layers like two-factor authentication and multi-signature transactions^64^. Research on the economic incentives and potential impact of double-spending attacks has led to the developing enhanced security protocols, promoting trust and reliability in cryptocurrency transactions.

#### Blockchain security and trust

Blockchain security and trust are critical considerations in designing and operating blockchain networks. The occurrence of 51% of attacks represents a significant threat to the security and trustworthiness of these networks. Extensive research has been conducted to assess the impact of such attacks on transaction integrity and overall reliability^65^. Successful attacks will compromise the accuracy and reliability of blockchain technology. Controlling network-mining power will lead attackers to manipulate the transaction history. This will cause double-spending attacks and confirms the earlier transactions^66^. This action will destroy the confidence of the user in the blockchain network. This will make users stumble on network transactions and cause fraudulent or fearful activities. However, the blockchain network always depends on user participation and adopting successful transactions, which causes economic repercussions^67^.

Earlier studies discussed the significant consequences of these attacks and recommended the importance of security measures to protect^68^. In addition, researchers are also focusing on mitigating and detecting 51% of attacks, which improves consensus mechanisms, decentralization governance, and enhancing network resilience^69^. Addressing the security and trust issues requires multi-faceted approaches. This approach involves technical solutions, regulatory measures, governance frameworks, and other industrial standards^70^. Research collaboration with policymakers, industrialists, and stakeholders ensures effective practices and security measures. Educating users and stakeholders will establish trust, comprehensive adoption, and usage.

#### Countermeasures and prevention

Hybrid consensus protocol combines PoW, PoS, and other mechanisms to improve security and avoid these attacks. These models will leverage the strengths of the approaches and mitigate the weaknesses. For instance, Hybrid PoW and PoS will improve security and reduce the mining centralization problem, which addresses the nothing at stake in PoS^71^. Likewise, hybrid PBFT leads to an increase in fault tolerance and scalability. These models can improve security and avoid 51% of attacks^72^.

Improving mining decentralization is also another preventive measure that has been proposed by researchers^73^. Promoting a distributed network of miners may lower the mining concentration power, which makes it more difficult for single or group entities to control the computational resources^74^. This technique can mitigate the problem of 51% of attacks through power distribution among various participants and confirms diverse and resilient networks. Introducing penalties for adverse behavior is vital to discouraging and avoiding 51% of attacks^75^. Consensus algorithms introduce various mechanisms that reduce the probability of 51% of attacks. However, improving network monitoring and malicious detections are essential to identify potential attack patterns, which trigger timely responses and avoid 51% of attacks. In machine learning, anomaly detection approaches are used to analyze the data and help detect suspicious activities that show the potential 51% of attacks^76^. Active network monitoring leads to the detection of attacks and ensures the security and integrity of the blockchain.

#### Impact on decentralization and consensus

The 51% of attacks can cause severe implications in the decentralization of the blockchain network. Hence, it should be considered as an immediate concern. Extensive research provides an understanding of the consequences of attacks on network governance, concentration power, and decision-making processes^77^. Decentralization shows that any single or group of entities does not control the network, immutability, resistance, and fostering transparency. However, a perfect 51% of attacks can allow an attacker to control the network and threaten decentralization. This power concentration contradicts decentralization, which introduces vulnerabilities and compromises the blockchain network's trust and integrity^78^. The existing consensus algorithms show the consensus reaching and validating the transactions*.* When a 51% attack occurs, the attacker can manipulate the consensus process, potentially invalidating transactions or reversing confirmed blocks. This disrupts the integrity of the consensus mechanism and raises concerns about the validity of the entire blockchain^79^. Research on the impact of 51% of attacks emphasizes the criticality of maintaining a decentralized network structure and robust consensus mechanisms. Efforts are directed toward developing countermeasures that promote decentralization and enhance the resilience of consensus protocols against such attacks^80^. Hybrid consensus models, for example, aim to combine multiple consensus algorithms to mitigate the vulnerabilities of individual approaches and achieve a more balanced and secure network. Safeguarding decentralization and consensus mechanisms also involves addressing factors such as governance and decision-making processes. Research explores ways to ensure fair and democratic governance structures where decision-making power is distributed among network participants^81^. Decentralized governance models prevent authority concentration with on-chain voting and transparent protocols.

In the real world, 51% of attacks serve as valuable resources for understanding the methodologies, impact, and responses associated with such attacks. Previous research has analyzed notable incidents, including attacks on Bitcoin Gold, Verge, and Ethereum Classic, to gain insights into the nature and consequences of these attacks^82^. By examining case studies, researchers can delve into attackers' specific techniques to gain majority control over the network's mining power. This analysis helps identify vulnerabilities within the consensus mechanisms and highlight areas where improvements are needed.

In recent years, many case studies have provided insights about the impacts of these attacks in the blockchain community, corresponding responses, and steps to reduce their effects^83^. Understanding the successful 51% of attacks can help to assess the economic losses, potential damage, and disintegration of user trust. This can be crucial for analyzing network participants to represent vulnerabilities, strengthen network security, evolve countermeasures, and provide active security strategies. Previous studies on 51% of attacks can provide the fundamentals to improve security, governance framework, developing mechanisms, monitoring, and detecting systems^84^. By gaining knowledge through these case studies, we can identify vulnerabilities, patterns, and best practices to develop more resilient blockchain networks. It is necessary to understand that the process of 51% of attacks can change frequently, leading to new attack vectors and the emergence of other techniques. Therefore, ongoing research and collaboration are essential to anticipate threats and address vulnerabilities associated with these attacks. The insights from case studies and real-world examples highlight the importance of continuous research efforts and interdisciplinary collaboration among researchers, developers, policymakers, and industry stakeholders. All these factors are combined to enhance security, trustworthiness, and resilience in the face of 51% attacks and other emerging threats to blockchain networks.

Consensus algorithms have several limitations that must be addressed to implement them effectively in real-world applications. Scalability is a significant concern, as many consensus algorithms need help to handle high transaction volumes and large network sizes. Finding efficient solutions to scale while maintaining security and decentralization is crucial for accommodating the demands of real-world applications. Energy efficiency is another limitation, especially in consensus algorithms like Proof of Work (PoW) that consume substantial energy. This not only makes them environmentally unfriendly but also economically unsustainable. Developing energy-efficient consensus algorithms is essential to reduce the carbon footprint associated with blockchain technology and ensure long-term viability.

Specific consensus algorithms introduce centralization risks, such as Proof of Stake (PoS) and Delegated Proof of Stake (DPoS). These algorithms can concentrate control in the hands of validators with more significant stakes or selected delegates, compromising the decentralization and trust that blockchain technology aims to provide. Balancing decentralization and stakeholder influence is crucial to maintaining a robust and inclusive network. Trust assumptions in consensus algorithms pose another limitation. Many algorithms rely on pre-approved validators or trusted authorities, which may not align with blockchain technology's decentralized and trustless nature. This limitation restricts the applicability of consensus algorithms in specific real-world applications that require higher levels of trust and security without relying on centralized entities.

Privacy and confidentiality are challenging within consensus algorithms prioritizing transparency and immutability. Striking a balance between data privacy and transparency is a complex task that consensus algorithms must address to protect sensitive information while maintaining the auditability and transparency required by various applications. Addressing these limitations in consensus algorithms will require continuous research, innovation, and collaboration between stakeholders in the blockchain community. By overcoming these challenges, consensus algorithms can unlock the full potential of blockchain technology in a wide range of real-world applications while ensuring scalability, energy efficiency, decentralization, trust, and privacy.

## Machine learning techniques for security enhancement

### Overview of machine learning algorithms applicable to blockchain security

Machine learning algorithms offer a range of techniques that can be applied to enhance blockchain security. These algorithms leverage artificial intelligence and data analysis to detect anomalies, identify patterns, and make predictions, thereby strengthening the resilience of blockchain networks against potential security threats. This section discusses an overview of machine learning algorithms applicable to blockchain security.

#### Supervised learning algorithms

Supervised learning algorithms, such as Support Vector Machines (SVM) and Random Forests (RF), play a vital role in blockchain security by enabling classification tasks and bolstering the detection and prevention of fraudulent or malicious activities^85^. Support Vector Machines (SVM) stand out as a widely utilized supervised learning algorithm in the context of blockchain security. SVMs excel in binary classification tasks, where transactions must be categorized as legitimate or malicious. By creating a hyperplane that separates the two classes in a high-dimensional feature space, SVMs strive to find the optimal hyperplane that maximizes the separation of data points while minimizing classification errors. SVMs boast a robust theoretical foundation and are particularly effective in scenarios where the data is not linearly separable. Kernel functions manage the high-handling feature spaces more efficiently and achieve linear and non-linear classification tasks*.*

RF is an ensemble learning algorithm that utilizes multiple decision trees and performs predictions^86^. Each decision tree in this algorithm is trained on the data subset and uses random feature selections. The final predictions can be made by combining the individual tree predictions. RF has the potential to handle high-dimensional data and its robustness. This technique is effective in handling tasks that involve regression and classification. In blockchain networks, RF is highly effective in determining whether the transactions are legitimate or fraudulent through investigating features and patterns related to their security. RF can also detect malicious nodes by analyzing their interactions and behaviors.

Both SVM and RF offer distinct advantages in terms of performance and interpretability. SVMs are acclaimed for their adeptness in handling complex data and finding optimal decision boundaries, while RF excels in managing large and diverse datasets^24^. Both algorithms can deliver accurate and reliable results within blockchain security applications. It is important to note that the efficacy of these supervised learning algorithms relies on the quality and representativeness of the training data. Labeled datasets containing examples of legitimate and malicious transactions or nodes are crucial for effectively training the models^38^. Furthermore, feature engineering plays a critical role in extracting meaningful features from blockchain data, thereby enhancing the performance of these algorithms.

#### Unsupervised learning algorithms

Unsupervised learning algorithms such as clustering and anomaly detection will aid blockchain security by identifying threats and patterns without labeled training data. Clustering algorithms group similar transactions or network entities in blockchain security. Algorithms such as k-means or DBSCAN partition the data into clusters, each representing a group of similar data points^42^. Clustering algorithms can differentiate between regular and unusual behavior by examining the patterns in each cluster. This helps to identify security threats or suspicious activities in the blockchain network. This capability is precious when specific attacks or anomalies are unknown in advance, as clustering algorithms can unveil unknown patterns or groups within the data.

On the other hand, anomaly detection algorithms focus on identifying outliers or anomalies that significantly deviate from the expected behavior^87^. Algorithms such as Isolation Forest or One-Class SVM construct models of the expected behavior within the data and flag any instances that fall outside this norm. In blockchain security, anomaly detection algorithms can help identify unusual or suspicious transactions, network nodes, or activities that may indicate potential security threats, fraud, or network intrusions^85^. Identifying these irregularities will lead to rapid implementation of security measures, minimize the risk, and protect the blockchain network's dependability. Unsupervised learning can also provide valuable insights into the character and structure of blockchain data. This makes it feasible to detect the potential security risks and irregularities that may not have been previously labeled or identified. These algorithms can provide a comprehensive and proactive approach that enhances the capabilities of blockchain security. An unsupervised learning algorithm requires expert parametric tuning to perform precise and significant outcomes^84^. In cluster algorithm selection, several clusters or thresholds for anomaly detection can significantly influence the algorithm's efficiency. However, data preprocessing and feature engineering are essential in unsupervised learning that prepares the data, which relies on inherent structure and data distribution.

#### Deep learning algorithms

Deep learning algorithms such as CNNs and RNNs significantly influence blockchain security^88^. CNNs are highly effective in analyzing structured data like transaction graphs. Meanwhile, RNNs excel in analyzing sequential data like transaction histories and brilliant contract execution. These algorithms can effectively identify malicious and fraudulent activities by detecting anomalies and patterns. It can provide advantages such as autonomous learning, accurate anomaly detection, and handling large datasets.

Moreover, deep learning algorithms can continuously improve their performance, allowing them to adapt to new attack patterns and evolving security threats^89^. Nevertheless, it is crucial to consider that deep learning algorithms require substantial amounts of labeled training data and significant computational resources to train and deploy effectively. Model interpretability and explainability can be challenging with deep learning models, given their complex nature and operation as black boxes. Ensuring the privacy and security of sensitive blockchain data during the training process is also a critical consideration^90^. By integrating deep learning algorithms, such as CNNs and RNNs, into blockchain security frameworks, researchers can enhance threat detection and strengthen the trustworthiness and resilience of blockchain networks.

#### Reinforcement learning

Reinforcement Learning (RL) is a valuable branch of machine learning that enables the training of intelligent agents to make sequential decisions to maximize cumulative rewards^91^. RL algorithms like Q-learning or DQNs can help create intelligent agents to secure blockchain with optimal security policies. Blockchain agents boost security by verifying blocks, selecting consensus methods, and preventing attacks. Through interactions with the blockchain network, RL agents observe the current state, take actions, and receive rewards or penalties based on the outcomes of their activities, thereby learning from their experiences during training^92^. RL agents explore the environment during training and learn through trial and error. The goal is to obtain a policy that increases rewards over time by enhancing security measures and reducing potential threats to blockchain security. RL empowers blockchain networks with adaptive and intelligent decision-making capabilities. RL agents can learn to identify attack patterns, anticipate vulnerabilities, and respond to emerging threats in real-time^93^. This adaptability is particularly valuable in blockchain security's dynamic and evolving landscape, where new attack vectors and vulnerabilities continually occur.

Furthermore, RL algorithms can be combined with other techniques like supervised or unsupervised learning to enhance the learning process. Pre-training RL agents with previous data or labeled examples improves their learning speed and performance in new situations. However, deploying RL algorithms in blockchain security presents challenges^94^. One critical challenge involves defining an appropriate reward structure that accurately reflects the security objectives of the blockchain network. RL agents must balance exploring new security measures and relying on established ones by finding a balance between exploring new actions and exploiting known strategies.

#### Bayesian networks

This algorithm uses probabilistic reasoning, which helps the system model and analyze the elemental correlations between the variables and blockchain security. It shows uncertain and incomplete information with directed acyclic graphs^95^. The calculations are performed for the event probability based on their observed evidence by quantifying conditional variable dependencies and probabilities. The Bayesian network provides valuable information, such as a blockchain network's security risks and vulnerabilities, and enables stakeholders to act appropriately^96^. By incorporating additional communication and handling missing or incomplete data with probabilistic reasoning, this algorithm is flexible and quickly adapted to changing the real-world scenario.

#### Generative adversarial networks (GANs)

In a blockchain network, GANs are a vital tool to enhance security in distributed networks. It has two components, a generator, and a discriminator, that create artificial datasets that replicate the real-world data exactly. GANs provide several benefits, such as creating diverse, realistic data for attacking scenarios^97^. This approach can simulate the attackers' actions and improve the network's defense system. However, this model can also enhance the anomaly detection system, improving blockchain security*.* In order to train the GANs, enormous computation resources and extensive training data sets are required^98^. Furthermore, it requires high-quality and diverse datasets, which influence the reliability of the synthetic data and improve the performance. Even though it has limitations, implementing GANs in blockchain provides real-world opportunities for developing attacking scenarios, testing blockchain security, and performing an effective anomaly detection system.

#### Privacy-preserving techniques (PPTs)

This technique has two security components, namely Differential Privacy (DP) and Secure Multi-Party Computation (SMPC). These components are necessary for maintaining confidentiality and anonymity in the distributed blockchain network^99^. DP can enable control over the noise, data perturbation, and query response, which avoids data point identification. However, SMPC works on multiple parts that collaborate with ML tasks and computations and improve privacy. Combining these approaches, PPTs can ensure the safety of the applicant's data and compliance with data protection regulations. Furthermore, it can also enable the detection of anomalies and prevent malicious activities^100^. While selecting the appropriate methods, we should consider the requirements such as privacy level, data type, analysis or prediction task, and computational overhead.

### Feature extraction and anomaly detection for 51% attack prevention

As a researcher, it is imperative to highlight the significance of feature extraction and anomaly detection in preventing 51% of attacks in blockchain networks. These techniques are pivotal in identifying and flagging abnormal patterns or behaviors that may signify potential attacks, allowing prompt intervention to mitigate associated risks. By delving into feature extraction and anomaly detection details, we can gain deeper insights into their crucial role in preventing 51% of attacks in blockchain networks.

#### Feature extraction

Analyzing blockchain data requires feature extraction, which is crucial in identifying and extracting meaningful information that captures the essential characteristics of transactions, network behavior, and participant activities^101^. Analysts obtain meaningful insights such as the blockchain network's security, performance, and efficiency, enabling information-based decision-making. Transaction-based features provide insights into individual transactions, encompassing transaction size, frequency, time stamp, input–output ratios, graph properties, and volumes^36^. Furthermore, these functions expedite the anomalies, detecting the malicious transactions and recognizing patterns associated with the malicious behavior.

The behavior, connectivity patterns, centrality, and consensus participation of blockchain are analyzed through network-based features. This analysis helps evaluate the network structure, identifies the significant nodes that influence it, and identifies any abnormal network activity^98^. Participant-based features center on individual participants within the blockchain network, encompassing reputation scores, stake sizes, and consensus participation history. These features assess the trustworthiness of participants, detect potential malicious actors, and evaluate the reliability of network contributors^102^. Smart contract-based features involve the analysis of smart contract code and properties, allowing vulnerability assessments, identification of attack vectors, and monitoring for suspicious behaviors during contract execution^59^.

Feature extraction is the foundation for subsequent analysis and modeling tasks in blockchain analysis. ML algorithms acquire the extracted features and perform statistical analyses. Researchers can gain insights that help to identify the pattern and make formal decisions based on these inputs. The feature selection always depends on the data under examination in blockchain and meets the goal, which helps the researchers better understand the blockchain network.

#### Anomaly detection

Detecting anomalies is critical, which secures the blockchain and maintains integrity. The process involves the identification of deviations that lead to potential attacks, abnormalities, and fraudulent activities. Irregularities can be identified using graph-based methods, behavioral analysis, ML, and statistical techniques^103^. Statistical approaches utilize statistical techniques to identify abnormalities in transaction patterns, network behavior, or consensus parameters. Clustering techniques group similar transactions or network entities to identify outliers or unusual patterns^98^. Outlier detection methods focus on identifying data points that significantly deviate from the expected distribution. Time-series analysis detects anomalies in temporal patterns or trends, enabling the identification of abnormal behaviors over time.

Machine learning approaches are impressive for anomaly detection in blockchain networks. Unsupervised learning algorithms automatically identify anomalies based on patterns and statistical deviations from normal behavior. These algorithms learn from previous data, detect subtle changes, and adapt to evolving attack patterns. Supervised learning algorithms can also be utilized with labeled data to classify instances as normal or anomalous based on known patterns^23^. Graph-based approaches leverage the structure of the blockchain network to identify anomalies. Graph analysis techniques detect changes in node centrality measures, unexpected or suspicious connections, or alterations in community structures. Analyzing the network graph can detect malicious activity like changes in consensus participation or new connections^36^.

The behavioral analysis focuses on establishing normal behavior profiles based on previous data. Analyzing transaction patterns, network interactions, or consensus participation can establish a baseline of expected behavior^104^. Deviations from these profiles, such as sudden changes in transaction volumes, irregular consensus participation, or unusual network interactions, are flagged as anomalies. Threshold-based approaches involve setting thresholds for specific features or behaviors and monitoring deviations beyond those thresholds. For example, exceeding a certain threshold in transaction volume or encountering a predetermined limit of consensus failures may indicate an anomaly^105^. These approaches are straightforward to implement and provide an early warning system for potential anomalies. By utilizing these approaches, researchers can enhance the security and robustness of blockchain networks by promptly detecting and mitigating anomalies.

#### Real-time monitoring and alerting

Real-time monitoring and alerting are integral components of a robust anomaly detection system in blockchain networks. The ability to detect anomalies in real-time and promptly respond to potential cyber-attacks is necessary for upholding network security and integrity^90^. Real-time monitoring ensures that any abnormal behavior or suspicious activities are swiftly identified and addressed, thereby minimizing the potential effect of attacks. Continuous analysis of blockchain data through ongoing anomaly detection methods is essential for real-time monitoring. As discussed previously, machine-learning models can be trained to detect anomalies in real-time and deployed to analyze incoming data streams for deviations from normal behavior or expected patterns^84^. The system uses machine-learning algorithms to identify and alert users of potential attacks or malicious activities as soon as they are detected. Automatic alerts are challenging to identify quickly and immediately respond to anomalies. This information can be sent through email, SMS, or mentioned on a dashboard. Integrating the ML model with the defined framework process can lead to real-time monitoring. Developing well-defined responding mechanisms involving active protocols isolating the compromised nodes and enabling additional security measures is necessitated^106^.

#### Dynamic adaptation

Dynamic techniques lead the blockchain network to adopt 51% of attacks that detect and respond to emerging threats. The continuous learning process in ML models can analyze the attacking pattern, detect new threats, prevent potential attacks, and improve accuracy^107^. This never-ending learning makes the system practical for identifying potential security risks. Feedback loops are critical for effective functioning. It can provide valuable information about false positives and negatives, improving anomaly detection accuracy, refining thresholds, and enhancing overall performance^100^. Dynamic adaption made the adjustments by consensus parameters or security measures and mitigated the potential attacks based on anomaly detection. Network configuration can be modified, limiting the action of malicious transactions or nodes and preventing possible attacks. Feature extraction is a critical factor in enabling dynamic adaptation and minimizing the effects of the attacks^108^. The system can identify the characteristics by extracting valuable information from blockchain transactions. This is more valuable to detect anomalies and identify deviations from the patterns. This technique will be updated based on new attacks and includes the latest indicators and behaviors related to 51% of attacks.

### Machine learning-based consensus decision-making

ML-based consensus hybridization is a promising method that enhances blockchain networks' security and trust. ML Techniques such as data analysis and pattern recognition can improve the network mechanisms. Intelligent decision-making is performed by leveraging ML algorithms. By investigating the previous data, ML models can collect the essential information that predicts the effects of various consensus performance parameters. This leads to optimizing the consensus parameters such as block size, difficulty level, and adaption over the change in network and improving scalability. In addition, ML-based consensus decision-making commits to blockchain security by implementing the identification, detection, and mitigation of suspicious attacks. ML models monitor the consensus process, detect anomalies, perform more proactive measures, and serve the network's integrity. Furthermore, this method also attains the potential to recast the consensus approach toward data availability, interpretability, privacy, and governance. This section discusses the different factors and their influence on the ML-based consensus decision-making process.

#### Consensus parameter optimization

Consensus parametric optimization is required to develop a high-performance, secure, and scalable blockchain network. ML algorithms optimize these parameters using the previous data and real-time network conditions. These models can investigate the previous data, which helps to identify the patterns and trends of the network performances through block size, difficult adjustment, time, and validation rules. ML model can identify the different parameters affecting the network's performance and optimize values that improve the efficiency of blockchain operations. In real-time scenarios, monitoring networking conditions allows ML models to adopt the dynamic consensus parameters, which examines the network metrics and provides necessary modification over the consensus mechanisms. These models provide valid suggestions to increase the block size and decrease block time, enhancing scalability in higher transactions. However, when the network faces any security threats, the proposed model learns from experience and recommends changing the difficult level or other validation rules and adapting consensus parameters to improve security. The proposed model can analyze the previous data and understand how the parametric setting affects the network performance and behavior. This learning model will refine the proposed system, which will result in improving the accuracy and optimizing the performance. Through ML, optimizing consensus parameters can improve network performance and security while also improving blockchain systems' scalability and adaptability. ML models can adjust the consensus parameters as the network changes to fit the new conditions, workloads, and threat levels. Adaptability allows the blockchain network to maintain its efficiency, security, and resilience even when faced with dynamic and changing environments.

#### Adaptive consensus selection

Blockchain networks need a suitable consensus algorithm that adapts to their dynamic conditions and needs. By analyzing past data and network metrics, ML is vital in choosing the best consensus algorithm. ML models can analyze performance metrics of various consensus mechanisms, such as transaction throughput and confirmation latency. These analyses can identify patterns and trends that highlight the strengths and weaknesses of each algorithm in specific network environments. Real-time monitoring allows the machine learning models to make adaptive decisions regarding consensus algorithm selection. The models constantly analyze network load, security needs, and node capabilities to select the best algorithm for the current situation. For instance, during heavy traffic, the system may choose a consensus algorithm that increases transaction speed. However, if security is the top priority, a Byzantine fault-tolerant algorithm may be selected instead. The ML models learn from previous data and adapt to changing conditions, improving their decision-making accuracy. Adaptive consensus selection brings numerous benefits to blockchain networks. Our system carefully selects the best consensus algorithm for every situation to maximize network efficiency, ensuring top-notch performance and resource utilization. Another important aspect is that it boosts network security by adapting the consensus algorithm according to the current security needs and potential threats. The network can protect itself against attacks and preserve the integrity of the blockchain system by adjusting to the situation.

#### Intelligent block validation

Ensuring the safety and accuracy of a blockchain network relies heavily on intelligent block validation. ML models can play a crucial role in this process. ML algorithms can help to make informed decisions about block validation by analyzing transaction data, network behavior, and consensus rules. By analyzing validated blocks from the past, these algorithms can detect patterns that signify valid transactions. This helps them develop a thorough understanding of a legitimate transaction. ML models categorize the valid and invalid blocks, improving block validation. This procedure can reduce manual inspection and detect malicious activities, improving efficiency in data transactions and network behavior. ML models can correctly identify potential threats, which leads to learning and evolving from the newly arrived data. This introduces effective adaption, changes the network conditions, and elevates themselves from attacking strategies. Furthermore, intelligent block validation improves security and confirms the valid blocks added to the blockchain.

#### Fraud detection and prevention

Blockchain networks can utilize the ML model and sophisticated algorithms, which analyze transaction patterns and network behavior and detect and prevent malicious activities effectively. The ML model analyzes previous data and detects unusual patterns and behaviors identified as fraudulent activity precisely and accurately*.* This makes the ML tools more powerful to maintain security and integrity. The proposed system uses supervised learning approaches and trains on labeled data to accurately classify whether the transactions are valid. This method also considers parameters such as transaction amount, time stamps, network interaction, and user behavior for exact predictions. Unsupervised learning methods can analyze the transaction pattern and network behavior, identify deviations from the normal ones, and finally detect anomalies in the blockchain data. This method detects fraud and unknown practices more effectively and identifies the double-spending or Sybil attacks as fraud attempts*.*

Furthermore, Natural language processing (NLP) techniques are used to analyze the textual data, feedback, and forum discussions to detect potential vulnerabilities. Keeping ML algorithms up-to-date and learning continuously from the new data will always recognize the patterns and prevent fraudulent activities. Earlier detection can reduce potential damage and enable high security. Hybridization of ML and Blockchain develops the network into more trustworthy and resilient.

#### Predictive analytics for consensus optimization

Predictive analytics are essential and enhance consensus mechanisms. ML models can analyze previous data and patterns, forecast network conditions, and optimize consensus processes. This model goes through different situations and predicts the network behavior by examining network latency, resource usage, and transaction throughput. This leads to the proposed model's ability to perform proactive, intelligent decision-making, which optimizes the consensus mechanisms. Predictive analysis can offer intelligent parametric optimization and adaptive consensus selection, which is anticipated to mitigate security threats. This allows the proposed system design to perform efficient and adaptable operations of the blockchain network under dynamic workloads and varying environments. Predictive analytics is useful for detecting and preventing fraud. Machine learning models can identify patterns of fraudulent activity, which can then trigger alerts and preventive measures for participants in the network. This proactive approach helps maintain the blockchain network's integrity and trustworthiness.

## Research methodology and materials

This section will discuss the proposed architecture's steps, components, and modules. The proposed architecture is developed using the ProximaX blockchain infrastructure platform that combines blockchain with the distributed service layers. This integrates blockchain networks with decentralized storage, database, streaming, and enhanced smart contract services to create an all-in-one user-friendly platform. ProximaX is designed to achieve high scalability, and throughput provides low latency. This platform is available in private, public, and consortium configurations and accommodates additional services without compromising the performance. This unique platform is built on reliable technologies that can be used in all industry segments. Researchers and practitioners can easily design and develop an application on a secure with high availability at a low cost.

**Figure 2:**
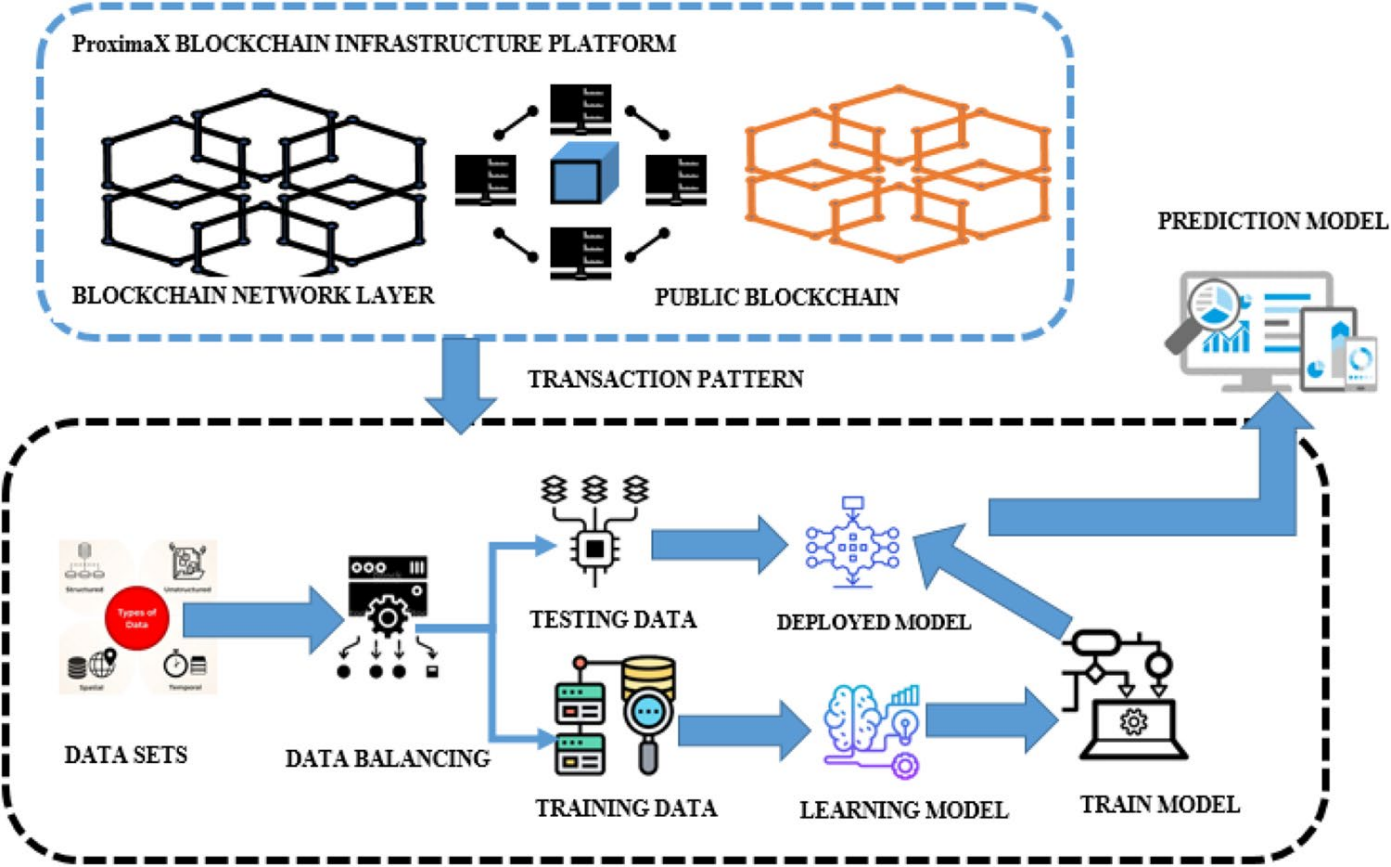
Proposed system using blockchain and machine learning layer.

The proposed architecture combines ML approaches with consensus to achieve agreement in a blockchain network. Integrating ML algorithms with consensus mechanisms can significantly enhance decision-making in distributed systems. These hybrid algorithms aim to address the limitations of consensus protocols by utilizing ML models. The algorithm gathers important data, extracts meaningful features, and trains ML models with data from the system model. These models are then incorporated into the consensus protocol to optimize decision-making, improve security through anomaly detection, and enable adaptive learning. Figure 2 provides a visual representation of how ML models are integrated with hybrid consensus algorithms in a ProximaX blockchain network to enhance efficiency, scalability, and fault tolerance while ensuring the integrity of the consensus process through the prediction model.

**Figure 3:**
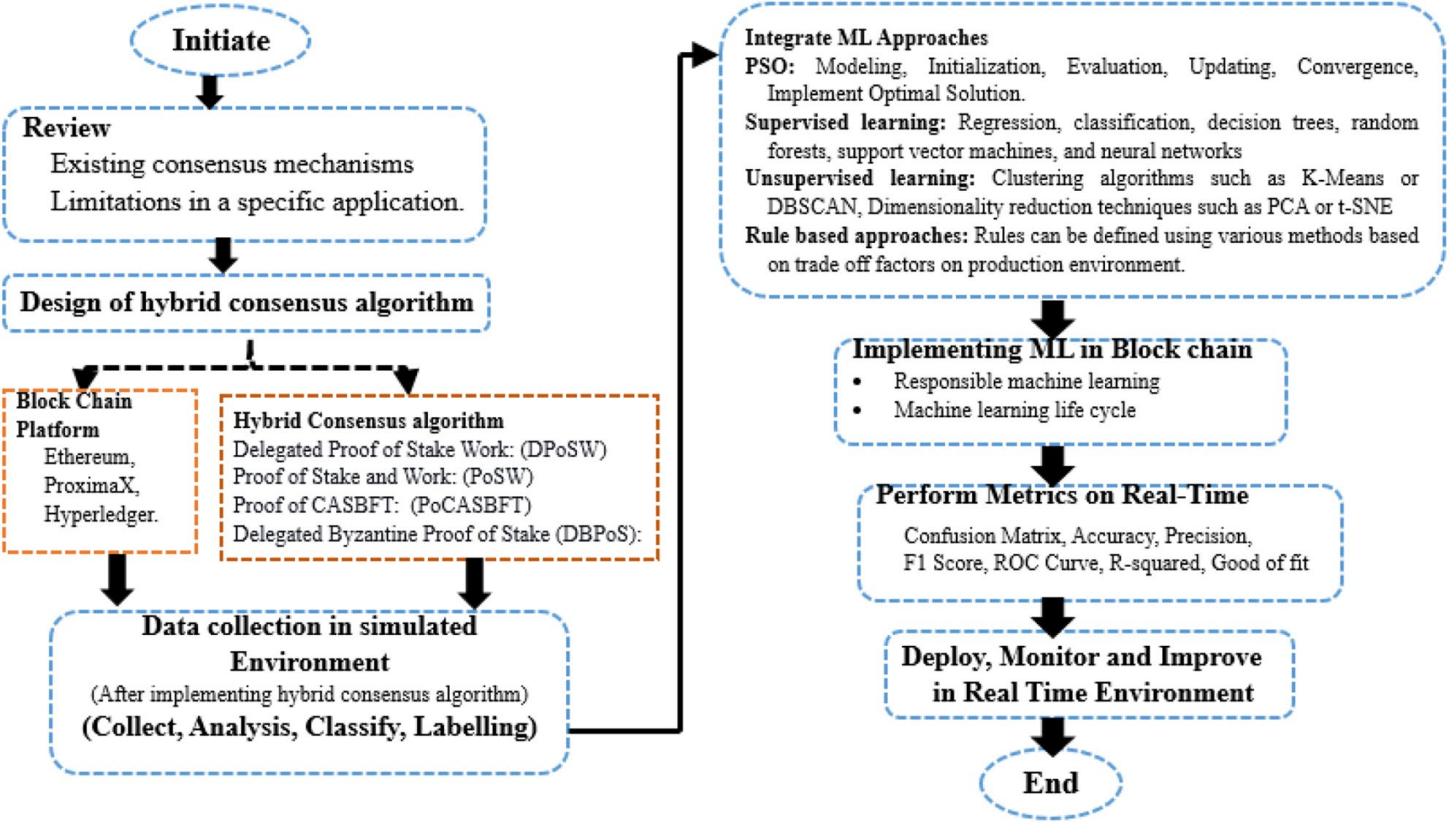
Design flow diagram for the proposed work.

Initially, the module is developed for collecting and extracting the necessary information from the ProximaX blockchain network. Next, the feature extraction module processes the data to extract meaningful features that capture pertinent information for consensus. The ML training module then uses various algorithms to train models based on the extracted features. The anomaly detection module analyzes incoming data using the trained ML models to identify abnormal behaviors or attacks. If any anomalies or attacks are detected, the consensus decision-making module evaluates them, assesses their impact, and determines appropriate actions to maintain consensus integrity. Finally, the consensus enforcement module ensures that the decisions made by the consensus decision-making module are enforced within the network. This iterative process involves continuous feedback, where data is collected, features are extracted, models are trained, anomalies and attacks are detected, consensus decisions are made, and enforcement is carried out. This enables the consensus architecture to adapt to changing network conditions, identify anomalies or attacks, and maintain consensus integrity based on intelligent ML decision-making. Figure 3 shows the design flow of the proposed work. The detailed discussion over the methodology involved in this research is discussed below through step-by-step analysis.

*Review and identify the attack scenarios*: The first step is reviewing existing consensus mechanisms and analyzing their limitations in required applications. Additionally, it identifies the different attacks that can occur in an application using blockchain. This information can be used to create a set of labeled data that can be used to train the machine learning algorithms.

*Choose a consensus algorithm*: The second step is to choose an appropriate consensus algorithm that meets the specific requirements of the blockchain-based system. Here, the ProximaX-based blockchain environment is considered. Hybrid consensus algorithms, which combine elements of different consensus algorithms, can provide robustness and security to the blockchain system. This can help prevent cyber-attacks, such as 51% of attacks and double-spending attacks. This algorithm will ensure the integrity and reliability of the blockchain. Some of the hybridizations of consensus algorithms discussed in this research work are listed below.

- *Delegated proof of stake work (DPoSW)*: DPoS validates blocks in the blockchain while PoW creates them, making it harder for attackers to manipulate the network. It uses a limited number of elected validators, enabling faster block confirmation times and higher transaction throughput than PoW. However, DPoS comes with the risk of centralization and relies on trust in elected representatives, which can compromise decentralization. These factors should be considered when evaluating consensus algorithms.
- *Proof of stake and work (PoSW)*: Blockchain uses PoS and PoW to enhance security and efficiency. PoW increases security against attacks, while PoS allows for better energy efficiency and scalability. However, PoS can lead to power concentration, requiring mechanisms to address the "nothing at stake" problem. Balancing decentralization and efficiency involves trade-offs. PoS creates governance complexity and requires careful management for transparency and inclusivity. Long-term security is a consideration as reliance on PoS increases and PoW decreases.
- *Proof of CASBFT*: Casper-PBFT is a hybrid consensus algorithm that combines Proof of Stake (PoS) and Practical Byzantine Fault Tolerance (PBFT) to improve network security and transaction speed. It offers strong consistency, rapid transaction finality, and scalability advantages. However, careful selection and governance of validators are necessary to avoid centralization risks, and the initial stake distribution may lead to power imbalances. Proper design, testing, and maintenance are necessary due to increased complexity. Adoption should be based on network requirements and associated trade-off management.
- *Delegated byzantine proof of stake (DBPoS)*: A secure and scalable system is achieved through a DPoS-PBFT hybrid algorithm. DPoS elects delegates for faster block confirmation and increased transaction throughput, while PBFT enhances resilience against failures. However, DPoS's small delegate set may lead to centralization and collusion risks, requiring proper governance and transparency. Careful design and monitoring are necessary to balance decentralization and efficiency. DPoS may affect decentralization compared to PBFT.

*Choose machine-learning algorithms*: Select ML algorithms, such as supervised, unsupervised, or rule-based learning, suitable for detecting and responding to attacks.

*Steps involved in ML*: ML approaches can be used to help prevent attacks on blockchain-based applications by:

- *Anomaly detection*: ML algorithms can identify and flag unusual network behavior, allowing network participants to quickly detect and respond to potential attacks.
- *Prediction modeling*: Predictive models can be trained to identify the likelihood of an attack based on previous data, allowing network participants to take preventative measures proactively.
- *Clustering and classification*: Clustering and classification algorithms can be used to identify and categorize different attacks, making them easier to understand.
- *Network traffic analysis*: Machine-learning algorithms can analyze network traffic and identify patterns that indicate potential attacks, allowing network participants to respond quickly.
- *Blockchain data analysis*: Machine-learning algorithms can be used to analyze the data stored on the blockchain, such as transaction history and network activity, to identify potential attacks.
- *Fraud detection*: ML algorithms can detect fraudulent transactions, such as double-spending or fake transactions, and prevent them from being added to the blockchain.
- *Risk assessment*: ML algorithms can be used to assess the risk posed by different nodes on the network and prioritize security measures based on the risk level.
- *Reinforcement learning*: ML algorithms can learn from network interactions and optimize the security measures to respond to potential attacks.

*Data collection*: Collect data from the required system to train the machine learning algorithms. This data should include normal and abnormal behavior patterns.

*Train machine-learning models*: Collect and label data from the system to train machine-learning algorithms, such as supervised, unsupervised, or rule-based learning, to detect and respond to cyber-attacks. This will help the algorithms identify abnormal behavior patterns that indicate an attack.

*Integrate the consensus algorithm and machine learning models*: Integrate the consensus algorithm and the machine learning models into the blockchain-based system such that the machine learning algorithms can identify and trigger a response mechanism through the consensus algorithm.

*Implementing ML in the blockchain*: Responsible machine learning (ML) involves developing, deploying, and using ML models that are ethical and accountable. This includes fairness, transparency, interpretability, and privacy. To achieve this, avoid biases and discrimination in data collection and model training, document decisions and assumptions, interpret predictions, and implement privacy safeguards. The ML life cycle includes formulation, acquisition, development, testing, deployment, and ongoing monitoring and maintenance. Responsible ML ensures the trustworthy and ethical use of ML technologies.

*Performance metrics*: To evaluate a machine-learning model, use metrics like Confusion Matrix, Accuracy, Precision, F1 Score, R-squared, ROC Curve, Area under ROC Curve, and Goodness of Fit. Analyzing these metrics helps identify areas for improvement and determine if the model suits the production environment.

*Monitor the system*: Continuously monitor the proposed system application and update the machine learning algorithms and consensus algorithm to adapt to evolving attack patterns.

*Respond to an attack*: If an attack is detected, the machine learning algorithms will trigger a response mechanism that is determined by the consensus algorithm. The response mechanism may include limiting the attacker's access, rolling back the blockchain, or triggering a secure emergency shutdown.

*Test and evaluate*: Test the hybrid system in a controlled environment to assess its effectiveness in detecting and responding to cyber-attacks.

*Deploy*: Once the system has been tested and evaluated, deploy the hybrid consensus algorithm and machine learning approach in the real-world environment.

*Monitor and update*: Continuously monitor the system for performance and security and update the ML and consensus algorithms to ensure their effectiveness against evolving attack patterns. However, implementing a hybrid consensus algorithm with an ML approach requires choosing an appropriate consensus algorithm, training ML models, integrating the consensus algorithm and ML models, testing and evaluating the system, and deploying the system.

### Experimental results and discussion

**Figure 4:**
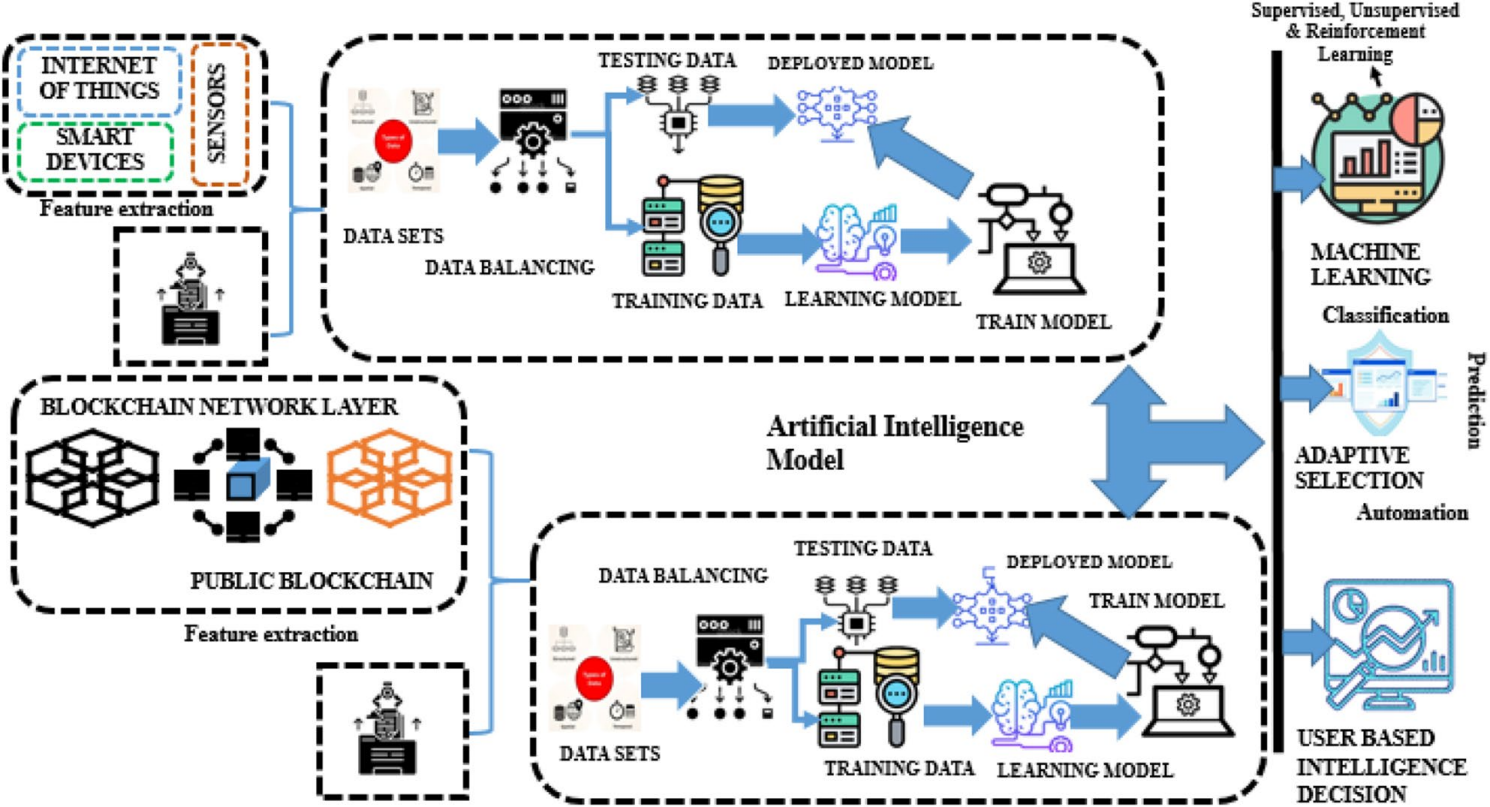
Experimental test setup with intelligence analysis.

Figure 4 shows the experimental diagram for this research work. In an IoT environment, obtaining vital information by deploying sensors that can detect various parameters from real-world scenarios is difficult. Establishing a robust system to collect this data in real-time or at regular intervals is paramount. Additionally, it is necessary to ensure the structured and balanced data and keep track of the time it was collected. Advanced techniques can be used to obtain valuable insights from the data. When training machine learning models, selecting and combining the most relevant features with labeled data is essential to building a training dataset.

Furthermore, correct labeling of the target variable for machine learning is necessary. When setting up a Blockchain network, it is essential to choose a proposed consensus algorithm that fits the network's needs, such as Delegated Proof of Stake Work (DPoSW), Proof of Stake and Work (PoSW), Proof of CASBFT (PoCASBFT) Delegated Byzantine Proof of Stake (DBPoS). This algorithm determines how nodes agree on transactions and adds new blocks to the blockchain. Collecting data from the network is required to detect potential threats. Information like transactions, block details, timestamps, and network states help us stay keen and secure. Relevant features related to transaction volume, block size, transaction fees, or other parameters should be extracted to identify potential attacks. Feature extraction techniques can analyze transaction patterns, mining activities, block propagation delays, or network connectivity measures. Anomaly detection algorithms, such as statistical methods, machine learning techniques, or graph-based analysis, can be used to identify abnormal patterns or behaviors. If an anomaly is detected, it should be labeled as a specific type of attack, such as double-spending, selfish mining, Sybil attacks, or 51% attacks. Ensuring equal representation of typical and attack instances in the training dataset is crucial to boosting the model's performance. This approach is necessary to prevent bias and establish the proposed model to achieve the user's requirement.

After preparing the model, it can be implemented into an IoT environment to analyze future data or make predictions in real-time. In order to make decisions fast and accurately, it is necessary to integrate the model coherently with the current IoT infrastructure**.** Meanwhile, deploying the trained model in the blockchain network can help to detect and monitor potential attacks in real time. Integrating the model into the blockchain infrastructure enables proactive defense mechanisms against malicious activities. After preparing the model, it can be implemented into an IoT environment to analyze future data or make predictions in real-time. Integrating the model with the existing IoT infrastructure is necessitated to ensure efficient and effective decision-making. To ensure strong security measures, continuously monitor and improve the model by gathering new data and adapting to emerging attack patterns.

Improving consensus algorithms' performance depends on factors like block confirmation time, transaction throughput, energy efficiency, latency, scalability, and fault tolerance. In this work, we can enhance the algorithm's performance by fine-tuning its parameters using optimization algorithms, grid search, or random search techniques. They can then evaluate the optimal parameter values using metrics that reflect the desired performance factors. By comparing different parameter configurations, we can choose the best values that maximize the desired performance factors. By implementing this methodology, we anticipate a more promising and enhanced efficacy within blockchain technology. In blockchain networks, various changes occur, such as network size fluctuations, workload, latency, or adversarial activities. An adaptive consensus algorithm that can adjust its behavior as needed is required to achieve optimal performance. Our work involves developing rule-based mechanisms that dictate the circumstances in which the algorithm should adjust. These guidelines can be determined by various factors such as network parameters, performance metrics, or system-level thresholds. By adhering to these conditions, the algorithm can adapt to changing network conditions by modifying its mode or parameters. Machine learning or reinforcement learning techniques can be leveraged for intelligent adaptive selection. By training models on previous data and network conditions, algorithms can autonomously adapt based on learned patterns or reinforcement learning rewards and penalties. It is necessary to consider the preferences and needs of the users during the adaptive selection procedure. Consensus algorithms are crafted by integrating user-specified criteria that align with the unique requirements of the blockchain application, including but not limited to scalability, security, and energy efficiency.

Understanding the needs of stakeholders, identifying the priorities, and considering specific applications are essential for developing an adoptable ML-based blockchain network. Integrating Consensus Algorithms with ML approaches attains the potential to optimize the network performance and effectively respond to the user's requirements. Stakeholders may possess transaction throughput, privacy, decentralization, energy efficiency, or consensus speed-based priorities. Analyzing these requirements will introduce intelligent selection, which selects hybrid consensus algorithms such as Delegated Proof of Stake Work (DPoSW), Proof of Stake and Work (PoSW), Proof of CASBFT (PoCASBFT), and Delegated Byzantine Proof of Stake (DBPoS). The proposed algorithms have their strengths and trade-offs in security, scalability, efficiency, and decentralization. However, these performance factors are determined by finding the required algorithms based on the user's requirements.

Optimization of the consensus algorithm requires the adjustment of the network parameters based on the user requirements. This can be achieved through several optimization techniques. Here, an ML-based blockchain network has been developed to monitor the network's performance and allows the system to make an adaptive decision based on the feedback from the real-world scenario. Continuous monitoring, evaluation, model refinements with periodic assessments, experimentation based on stakeholders' responses, and user feedback are highly required to implement successful models. Valuable insights are gained by performing the investigation using ProximaX, and results are expected to overcome the different types of attacks in the proposed work. The expected results are listed below.

- Real-time attack detection and response,
- Maintenance of blockchain integrity,
- The smooth operation of a proposed application,
- Adaptability to evolving threats,
- Improved security,
- Minimized attack damage,
- Efficient defense mechanism,
- Increased trust in the system, adoption, and better performance.

The prime objective of this research work is to identify and respond to the attacks in real-time scenarios immediately. This can be achieved by implementing the ML model to determine the paranormal behavioral patterns that act as a threat. This improves the security and resilience of the blockchain network and minimizes the damage. Maintaining the integrity of the blockchain network is another important objective. Consensus algorithms can validate the transaction and ensure the reliability and stability of the Blockchain network. By combining these Consensus algorithms with a hybrid approach, the proposed system attains higher transactional data integrity and lowers unauthorized activities. ML model and Hybrid consensus mechanisms are combined to ensure convenient operations. These results in highly comprehensive defense mechanisms. This approach can reduce the downtime risk and provide effective operations. However, this approach is adaptable to an evolving new threat and maintains reliable and efficient operations in a specific application-oriented system. The proposed ML models can be kept updated and customized to encounter emerging attacks, which performs the guaranteed defense mechanism for real-world environments.

This approach can proactively identify, detect, respond to, and mitigate the threats. However, it can also reduce the system vulnerability and damages caused by the threats. Expeditious measures are performed to protect the stability and functionality of the network, ensuring better system operations. The proposed hybrid approaches can efficiently protect the blockchain-based system against attacks. The proposed method adapts and updates continuously, which performs fine-tuning to understand emerging threats. This also enhances trust and encourages adaptability in intelligent grid sectors. These features can increase the stakeholder's confidence and improve system performance, resulting in stability, security, and efficient defense against attacks.

## Security enhancements achieved through the proposed solution

By combining ML techniques with consensus protocols, the blockchain network performs anomaly detection, adaptive decision-making, and detection of malicious activities. These techniques can detect and mitigate various cyber-attacks by continuous network monitoring and analyzing real-time datasets. However, this hybrid approach provides robust security enhancements that enhance decision-making. This section discusses the various security enhancements achieved through the proposed methodology.

*Increased attack resistance*: Incorporating ML techniques and hybrid consensus protocols in blockchain networks increases the attack's resistance. This identifies and prevents attacks such as 51%of attacks, Sybil, and double spending attacks. ML models can detect potential attacks by collecting transaction details, network connectivity, and participant behavior. However, the proposed method ensures network security and integrity. This ML model analyzes the network behavior and transaction patterns using previous data obtained from the network, which helps to detect malicious activities and perform precautionary measures. This can prevent almost half of the attacks, detect malicious mining behaviors, and immediately send alert information to prevent attacks. These proactive techniques will minimize the negative impacts of the blockchain network and maintain high security, which is appreciable for the stakeholders and network participants.

Furthermore, the ML model can prevent Sybil and double spending attacks on blockchain networks. These models can use the previous network information and detect irregularities, flags, and fraudulent activities. Combining ML algorithms with a hybrid consensus process will improve the network's ability, promoting confidence in the blockchain network and protecting the stakeholder's assets and transaction details.

*Dynamic threat detection*: ML algorithms play a crucial role in detecting new threats and identifying the attacking patterns in the blockchain network. The proposed ML model can identify the unusual behaviors and patterns that represent potential attacks by analyzing the datasets from the existing networks. In addition, continuous learning in ML techniques leads to the immediate adaption of new approaches used by malicious attackers, which develop the network into highly secure. These algorithms can detect attacks, including DDoS, Network intrusions, data breaches, and malware propagation. ML model can perform dynamic threat detection, which enhances blockchain security and promotes trust among the network participants and stakeholders.

*Anomaly detection and prevention*: Effective anomaly detection and introducing preventive measures lead to maintaining the high security and integrity of blockchain networks. ML models are irreplaceable tools that detect mischievous transaction behavior, network connectivity, and other participant activities. It can perform proactive detection and prevent suspicious activities. Analyzing transaction records, network logs, participant interactions, and other system parameters leads the ML model to learn the standard patterns and behaviors exhibited within the network. However, these models establish the expected baseline and detect the deviations from these patterns that indicate fraudulent activities. ML techniques such as clustering, outlier detection, time-series analysis, and graph-based techniques are used to identify potential attacks. Integrating these techniques with blockchain networks can enhance proactive identification, which detects malicious activities in real time.

Furthermore, anomaly detection can deter potential attackers and discourage fraudulent activities*.* This can improve the confidence level of the stakeholders and participants and promote the adoption of blockchain technology. The proposed ML model can integrate the network decision-making process. For instance, detecting anomalies leads the network to trigger additional verification automatically and employs priority-based security over efficiency. This adaptive response makes the system resilient, secure, and trustworthy to face potential threats.

*Adaptive decision-making*: ML models facilitate the blockchain networks based on previous data in real time and adapt the decision-making process. This systematic approach can improve performance and optimize security by investigating network congestion and resource availability. We can analyze the metrics and adjust the consensus parameters model with ML approaches. This leads to block time and size variations that improve the transaction throughput and network performance in congested networks. Similarly, for detecting malicious behavior, the proposed model suggests actions such as improving validation regulations and tweaking the consensus protocols. These models can be trained based on previous data, which performs the adaptive choices to enhance security. It can identify patterns or trends that affect network performance and security by analyzing experiences. This knowledge allows the models to make informed decisions and adjust the consensus parameters accordingly. Adaptive decision-making is essential for blockchain networks because it enables quick response to changes by adjusting real-time consensus parameters. It also enhances security by detecting and responding to emerging threats using ML models. This approach optimizes resource allocation, improving efficiency, scalability, and network participation. Continuously adapting decision-making strategies ensures the network remains responsive to emerging challenges and continually improves performance, security, and resilience.

*Robust network monitoring*: The safety and stability of blockchain networks heavily rely on dependable monitoring. Hybrid consensus protocols use ML to continuously monitor and analyze the network, detecting security threats, attacks, and irregular behaviors. ML algorithms are particularly effective at identifying patterns and anomalies in large amounts of data, such as traffic and transaction data. Monitoring the blockchain network in real-world scenarios provides earlier warnings and other appropriate trigger responses. Robust monitoring can detect critical behavior, potential attacks such as DDoS, Sybil, tampering with the data transactions records, and other security breaches. This also uncovers the security vulnerability and pattern with exploitable loopholes, mitigates the attacks, and maintains the network integrity. ML models address these issues proactively and improve the system's ability to fight security threats and enhance overall network security. Furthermore, these models can identify recurring patterns and adjust their response strategies to improve outcomes in strengthening high adaptability. By using federated learning and homomorphic encryption, consensus mechanisms can detect anomalies and security breaches, improving the privacy and security of the entire blockchain network.

### Scalability, decentralization, and energy efficiency considerations

The hybridization of consensus algorithms and ML models addresses challenges such as scalability, decentralization, and energy efficiency in distributed systems. This approach can disseminate the workload across various machines, perform effective data processing, and execute parallel algorithms. The decentralization process can be achieved using P2P networks, which improves reliability and prevents failures. To ensure data availability and integrity, redundancy and replication techniques like sharding or erasure coding can be used, even in node failures. Energy efficiency has become a critical concern as the demand for sustainable computing solutions grows. Consensus algorithms and ML models should be hybridized while considering energy efficiency to minimize environmental impact and operating costs. Use specialized hardware or cloud services to save energy when performing resource-intensive tasks like machine learning or complex consensus computations. These specialized infrastructures are designed to maximize computational efficiency, reducing energy requirements. Developing consensus algorithms with energy efficiency in mind can also minimize computational and communication overhead. PoS consumes less energy than PoW algorithms, relying on stakeholder voting rather than resource-intensive mining. ML models can be compressed or quantized using techniques like pruning, quantization, and knowledge distillation to reduce their size and energy consumption without sacrificing accuracy. Hybridizing consensus algorithms and machine learning models can address distributed systems' scalability, decentralization, and energy efficiency challenges. Considering these factors, we can develop sustainable solutions that leverage the best of both worlds.

### Advantages and optimizations achieved through the proposed approach

**Figure 5:**
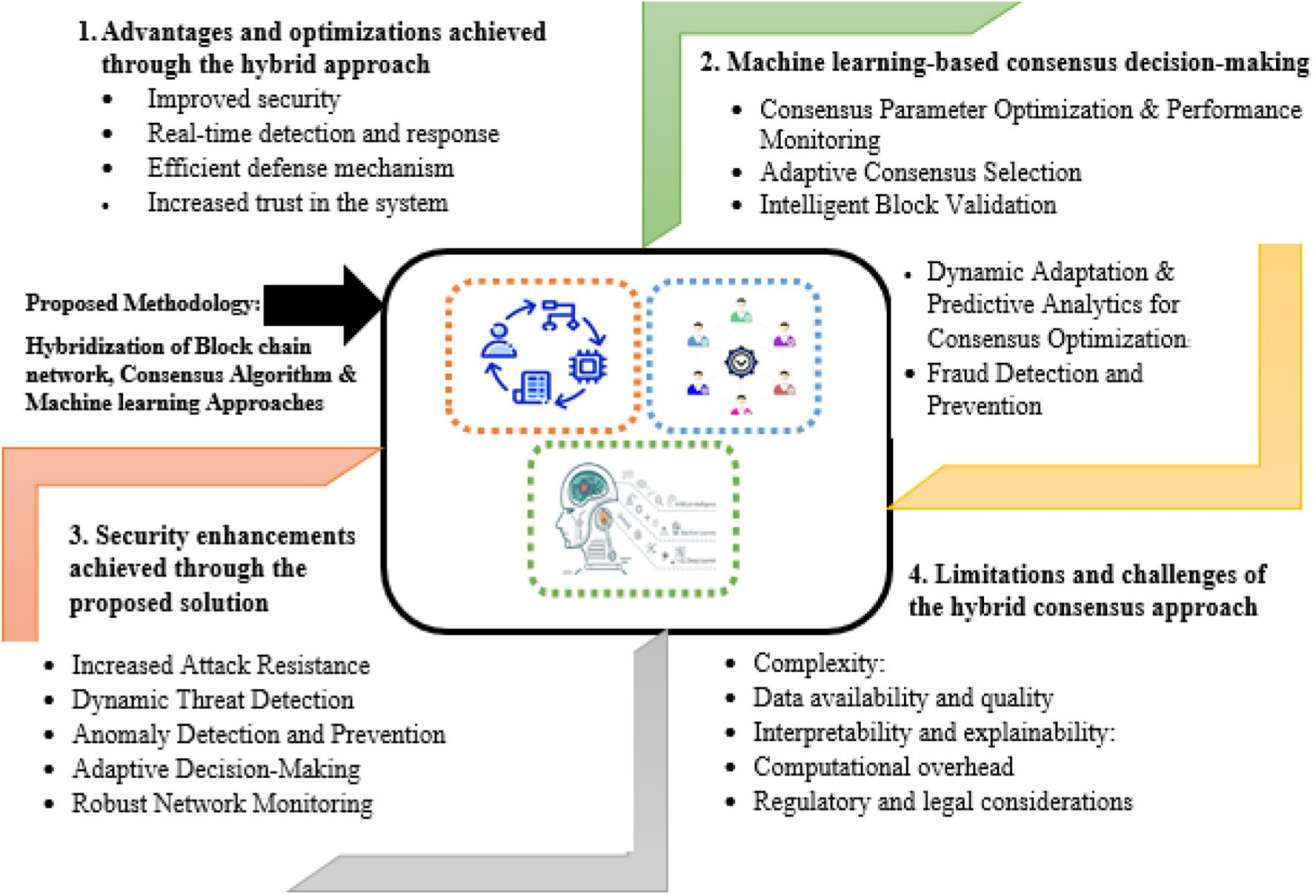
SWOT analysis of the hybrid approach.

Hybrid consensus algorithms and ML approaches are widely used to overcome various attacks in blockchain technology. The advantages and optimizations achieved through the proposed approaches are listed below. The key insights and SWOT analysis of the proposed research work are shown in Fig. 5

#### Improved security

Advanced security is necessary for safe and reliable blockchain network systems. Practical ML techniques with hybrid consensus create a secured network with high integrity and reliability, preventing potential attacks and negative impacts. This model can establish a trust-based framework that ensures the network nodes perform valid transactions and avoid unauthorized attackers. Furthermore, some attacks require other measures that represent abnormal behavior. ML techniques can investigate substantial data volumes, identify patterns, and detect malicious attacks in real time. Here, continuous monitoring of energy transactions, communication patterns, and other system parameters is performed by integrating ML techniques. The system can utilize the previous data and effectively identify the unusual activity of ongoing attacks. Combining consensus and ML algorithms can create a robust defense mechanism and prevent cyber-attacks. Integrating ML techniques leads the system to detect and prevent the attack before any damage. These techniques can adapt and improvise through continuous learning from new data and evolving attack techniques. This adaptability can improve the system's ability to detect threats and respond effectively to unknown attacks, strengthening security.

#### Real-time detection and response

Real-time detection over frequent responses is necessary to ensure the security and stability of the proposed systems. ML techniques are generally trained on the previous data and establish the baseline to understand normal behavior, energy consumption, communication patterns, system metrics, and other relevant data. By identifying the anomalies, ML techniques can provide the response mechanisms to isolate the affected nodes, reroute the energy transactions, and activate the alerts and backup systems for further investigation. For example, The proposed system with microgrid application can provide services to hospitals, emergency services, and remote community sectors, requiring real-time detection and responses and ensuring uninterrupted operations and reliability. ML techniques can identify the attacks in time, respond quickly to process the data and ensure the decision is based on learned patterns. This system can ensure security, detect real-time threats, and reduce the vulnerability window that causes minimum damage by utilizing ML techniques.

#### Evolving security

Evolving threats can be avoided using hybrid consensus and ML algorithms. Consensus algorithms can secure the blockchain, and ML techniques analyze various data sources and detect new attacks, adapting defense systems to ensure security. Data analysis tools can monitor parameters such as network traffic, transaction patterns, user behavior, and other system logs. These systems can analyze new data, improve their ability to detect suspicious activities and perform immediate alerts for further investigation. ML algorithms can perform collaborative acts and rely on information sharing for evolving defense mechanisms. Integrating consensus and ML algorithms can strengthen the synergistic effect and be updated regularly through ongoing research, collaboration, and information sharing within the blockchain community. This system can stay connected to the latest attack techniques, which helps to mitigate emerging threats and ensures long-term security.

#### Efficient defense mechanism

An effective defense system can be developed using consensus and ML algorithms that detect and respond automatically. In microgrid applications, consensus algorithms can provide authorized transactions that ensure integrity and reliability. ML algorithms can analyze the data, identify patterns, learn from previous attacks, and predict the detection. These ML models will monitor the microgrid system, identify network behavior, and analyze the transaction patterns. This leads to the proposed algorithm detecting potential attacks, identifying the deviations, evolving the triggering process, and mitigating their impacts on microgrid security. Combining hybrid consensus algorithms and ML techniques creates an effective defense mechanism that performs an immediate detection and response to any attacks on the microgrid. Through continuous learning, the ML model develops into an automation process that reduces the administrator's workload, enhances the efficiency of the defense mechanism, and enables real-time detection and response to attacks. These processes can be optimized through iterative learning approaches in ML techniques. Through the application of these methodologies, the proposed system has the potential to enhance the resilience and efficiency of microgrid systems*.* These algorithms can automate processes and continuously learn, which reduces the workload on system administrators and promotes a proactive approach to security. Ultimately, this helps to defend against attacks while ensuring the uninterrupted operation of the system.

#### Increased trust in the system

Trust and security are crucial in microgrid systems, and blockchain-based microgrid systems offer a solution that goes beyond. Combining hybrid consensus algorithms and ML approaches can enhance trust in the system's design and improve its adaptability. Using hybrid consensus algorithms and machine learning techniques creates a robust defense system to protect microgrids against potential attacks. This way, it establishes trust by verifying transaction validity and preventing malicious activities through consensus among network participants while also allowing for continuous learning from new data and emerging attack patterns through ML algorithms. Adaptability is of utmost importance in maintaining the effectiveness of defense mechanisms in microgrid systems, especially against constantly evolving and sophisticated strategies. Through ML algorithms, this hybridization can detect anomalies, predict real-time attack patterns, and adjust the defense mechanism accordingly. As a result, this instills increased trust in the microgrid system, enhances its resilience, and reduces its vulnerability to evolving threats.

## Open issues and challenges of the hybrid consensus approach

### Open issues of the proposed research approach

Integrating ML, deep learning, and RL with blockchain protocols can improve security, performance, and decision-making capabilities. However, it also presents open issues and challenges that researchers and practitioners must consider carefully. In order to discover the benefits of these technologies in blockchain networks, it is essential to understand and address these challenges. This research analyzes the challenges of integrating intelligent learning algorithms with consensus protocols in blockchain networks. However, it also explores the factors researchers and practitioners should consider to ensure blockchain networks' effective and efficient operation. This analysis identifies critical limitations such as machine-learning algorithms' computational complexity and resource requirements, the need for labeled training data in decentralized and pseudonymous blockchain networks, and adaptive learning approaches in dynamic blockchain networks. Investigations are performed to understand the significance of security and privacy in integrating ML techniques, which includes the vulnerability of models, malicious attacks, and other challenges that ensure interpretability in decision-making processes. However, it is observed that valuable insights into the difficulties of Implementing ML-based consensus algorithms in blockchain. Our findings can also provide future research and development efforts to address the practical concerns and overcome these limitations.

#### Complexity

Incorporating hybrid consensus algorithms with ML models will be challenging and complex. To achieve this, we need a strong understanding of consensus protocol, ML algorithms, and sufficient computational resources for training and executing the models. Coordinating the computational resources with consensus protocol and integrating the ML approaches into the hybridization are significant challenges in real-world scenarios.

#### Data availability and quality

Accurate training of ML models requires diverse and high-quality data. Nevertheless, this can be challenging in blockchain networks, where data privacy and confidentiality are crucial. Furthermore, obtaining labeled data for supervised learning requires specialized expertise and extensive manual work that can be time-consuming and expensive.

#### Model robustness and generalization

For developing successful hybrid consensus algorithms, the ML model must possess resilience and the capability to perform effective generalization in untested data. Inadequate adaptation to new attack patterns or overfitting can compromise the effectiveness of the models, leading to the identification of false positives or false negatives, which can ultimately weaken the entire system.

#### Interpretability and explainability

Understanding the reasoning behind ML models can be difficult, as they often function like mysterious black boxes. However, when it comes to consensus protocols, transparency and accountability are crucial, which means that methods must be employed to clarify the choices made by these models. This task can be incredibly daunting when dealing with complex models such as deep learning architectures.

#### Malicious attacks

It is challenging to be aware of potential threats to the machine learning models, such as false training data, harmful adversarial examples, and weaknesses in the learning process. These attacks can negatively affect the reliability and effectiveness of the hybrid consensus approach. Therefore, it is essential to implement robust defenses to minimize their impact.

#### Computational overhead

Incorporating ML models into the consensus process could increase the amount of computational work needed. Developing and implementing intricate machine learning models may demand substantial computational resources, which could affect the scalability and effectiveness of the blockchain network.

#### Ethical considerations

It is imperative to consider ethical implications such as privacy, fairness, and bias when utilizing ML models in consensus protocols. We must take precautions to prevent infringement on user privacy or discriminatory behavior towards certain participants due to biased training data or decision-making processes. Addressing these issues will lead to more effective and equitable use of ML models.

#### Regulatory and legal considerations

Applying ML models in consensus protocols may raise regulatory and legal challenges, especially in industries with strict data protection and compliance requirements. Compliance with data privacy regulations and ensuring the lawful use of data becomes crucial when integrating ML into consensus mechanisms. Addressing these limitations and challenges requires careful consideration of the specific use case, continuous research and development, and collaboration between domain experts in consensus protocols, machine learning, and cybersecurity. By addressing these challenges, the hybrid consensus approach with ML models can unlock its full potential in enhancing blockchain networks' security, scalability, and adaptability.

### Future research directions

- The future scope of this research leads to adaptive hybrid models, which focus on developing new consensus mechanisms and ML techniques. These combinations can dynamically adapt to networking situations. It also includes switching between other ML models or consensus mechanisms and responding to the security threats that evolve based on the scenario.
- Privacy-preserving ML techniques are essential for advancing research. These hybrid models effectively integrate homomorphic encryption, federated learning, and differential privacy. This ensures that the user's sensitive information remains secure and confidential while performing the learning process.
- Future research should focus on self-learning systems, which have the potential to hybrid consensus mechanisms. These systems can adapt autonomously to new threats and optimize the system parameters per the network conditions' real-time feedback. However, energy efficiency is the paramount factor in the blockchain network. Research efforts should focus on developing effective energy consumption-based consensus mechanisms and ML models.
- The security and the consensus mechanism's performance can be ensured by establishing security standards and benchmarks for the evaluation process. This facilitates comparing various approaches and contributes to developing new effective models. Cross-collaboration across experts in consensus algorithms, machine learning, cryptography, and cyber security leads to novel methods and insights to overcome real-world problems.

## Conclusion

In this research, we have thoroughly analyzed the hybrid consensus algorithm with ML techniques. The challenges and vulnerabilities that exist in a proposed system are concluded using a ProximaX-based decentralized network platform. The findings from this research paper have emphasized the need for effective preventive measures to combat the detrimental impact of cyber-attacks. The proposed research uses hybridized consensus approaches to enhance network security, scalability, and resilience. The proposed ML-based hybrid consensus algorithm mitigates the challenges and vulnerabilities in decentralized public networks. The ML model implemented in this research elevates threat detection, optimizes consensus mechanisms, and ensures the confidentiality of transactions and user data. The decentralized nature of ML-driven security performs proactive attack identification, feature extraction, and anomaly detection, enhancing the consensus protocol's security and reducing cyber-attacks. The proposed model understands the consensus mechanism and retrieves the real-time data and network state, which improves network resilience and decision-making accuracy in the dynamic field of cyber security.

Furthermore, this paper also discusses the implementation challenges of the proposed consensus approaches and their adaptability to a real-world scenario. Further investigation and refinements are required to solve the complexity of the ML model with scalability, resource requirements, computational overhead, and susceptibility to achieve the effectiveness of security and trustworthiness. The future scope of this research leads to the development of an adaptive hybrid model that utilizes novel consensus mechanisms and ML techniques. Privacy-preserving ML techniques and generative learning can autonomously adapt to new threats and optimize the system parameters based on the real-world environment.

## Data availability

The datasets used and/or analysed during the current study available from the corresponding author on reasonable request.
